# Diversity of Eastern North American Ant Communities along Environmental Gradients

**DOI:** 10.1371/journal.pone.0067973

**Published:** 2013-07-12

**Authors:** Israel Del Toro

**Affiliations:** 1 Organismic and Evolutionary Biology, University of Massachusetts Amherst, Amherst, Massachusetts, United States of America; 2 Harvard Forest, Harvard University, Petersham, Massachusetts, United States of America; Field Museum of Natural History, United States of America

## Abstract

Studies of species diversity patterns across regional environmental gradients seldom consider the impact of habitat type on within-site (alpha) and between-site (beta) diversity. This study is designed to identify the influence of habitat type across geographic and environmental space, on local patterns of species richness and regional turnover patterns of ant diversity in the northeastern United States. Specifically, I aim to 1) compare local species richness in paired open and forested transects and identify the environmental variables that best correlate with richness; and 2) document patterns of beta diversity throughout the region in both open and forested habitat. I systematically sampled ants at 67 sites from May to August 2010, spanning 10 degrees of latitude, and 1000 meters of elevation. Patterns of alpha and beta diversity across the region and along environmental gradients differed between forested and open habitats. Local species richness was higher in the low elevation and warmest sites and was always higher in open habitat than in forest habitat transects. Richness decreased as temperature decreased or elevation increased. Forested transects show strong patterns of decreasing dissimilarity in species composition between sites along the temperature gradient but open habitat transects did not. Maximum temperature of the warmest month better predicted species richness than either latitude or elevation. I find that using environmental variables as key predictors of richness yields more biologically relevant results, and produces simpler macroecological models than commonly used models which use only latitude and elevation as predictors of richness and diversity patterns. This study contributes to the understanding of mechanisms that structure the communities of important terrestrial arthropods which are likely to be influenced by climatic change.

## Introduction

Biodiversity monitoring studies along environmental gradients can be used as natural experiments to document how species richness and community structure change in response to biotic and abiotic factors, including those predicted to be affected by climatic change [1], [2]. The latitudinal gradient of species richness is well documented for multiple taxa and throughout many regions globally [3]. At regional spatial scales (i.e. scales within the same biome, domain or landscape [4]), species richness is often correlated with temperature, water availability, and productivity [5], [6], [7], which also vary with latitude. These correlations may differ among continents, perhaps reflecting differences in evolutionary history [8]. At local spatial scales (i.e. scales within the same community [4]), habitat type may be a better predictor of species richness than geographic location along an environmental gradient [7]. Both regional and local scale processes affect species richness when measured at various spatial scales [8], [9]. This work contributes to the growing number of studies relating species richness to various environmental gradients thorough different global biomes (e.g. [7]) and so can be useful in identifying global patterns of species richness.

Understanding regional patterns of species richness also requires an understanding of how species turnover changes across environmental gradients. Beta diversity analyses are necessary for identifying the environmental correlates that contribute to dissimilarity between local communities across regional scales. Beta diversity analyses explore the relationships between local and regional richness and ultimately help explain how communities assemble due to the influence of local and regional environmental filters [10], [11].

Changes in regional climate can modify the communities of organisms that inhabit the region [12], [13], [14]. If changes in community structure and composition associated with climate change are substantial and impact keystone and abundant species (which may be responsible for sustaining ecosystem processes and services), the changes in composition may lead to large ecosystem-level consequences [15].

In this study, I assessed how species richness of ants of the northeastern United States changes across environmental gradients. Ants were ideal organisms to use for such a study because they are locally abundant throughout the study region, their diversity in the region is relatively well understood, approximately 180 species occur in the full extent of the study region, and standardized sampling methods can be implemented easily and replicated at regional scales [16], [17]. Ants also provide key ecosystem services and mediate various ecosystem processes [15].

The main objective of this work was to document the patterns of ant species richness across 10° of latitude and ∼ 1000 m of elevation relief across the forests of the Appalachian Mountains in the northeastern United States. Specifically I: 1) compared local species richness patterns in paired open and forested habitat transects; 2) identified the environmental variables that best predict richness patterns; and 3) documented the patterns of beta diversity throughout the region in both open and forested habitat.

## Materials and Methods

### Study Region

**Figure 1:**
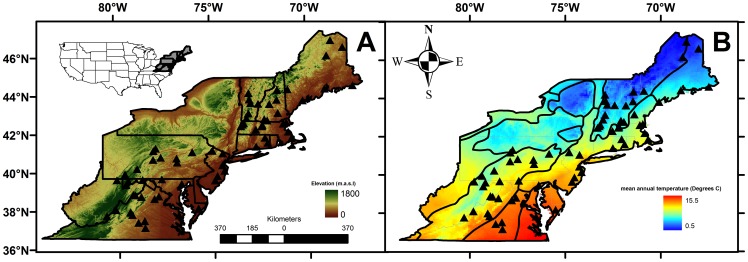
Sampling sites. Distribution of sampling sites across A) an elevation gradient with state boundaries and B) a temperature gradient in the Northeastern U.S. with EPA Level II ecoregion boundaries.

The forests of the eastern United States span approximately 1500 m in elevation relief across the Appalachian Mountain range (Figure 1A), which extends into the southeastern United States. I sampled ants at 67 sites spanning 10° of latitude across the northeastern United States (Figure 1). The sampled sites were distributed across five of the Level II ecoregions of North America [18] [19]: (1) Atlantic Highlands, (2) Mixed Wood Plains, (3) Southeastern U.S. Plains, (4) Ozark-Oachita-Appalachian Forests and, (5)Southeastern Coastal Plains (Figure 1B). In the extent of the study the highest site was at ∼1000 m.a.s.l. at Mount Greylock State Reservation, Massachusetts and several sites in Maine, Massachusetts, New Jersey, Maryland and Virginia were close to sea level. Both elevation and latitude influence the climate at each site. High elevation and high latitude sites are restricted to the Atlantic Highlands ecoregion; they have mean annual temperatures 4°C±3.2°C. Low elevation and low latitude sites were located in the Southeastern Costal Plains ecoregion and had a mean annual temperature of 13°C±1.2°C (Figure 1B). Site information, including the responsible permitting agency for all sampling sites is presented in (File S1). No endangered or threatened species were collected as part of this study.

### Ant Sampling

At each site I sampled ants using pitfall traps along two, 200 m linear transects. At each site, one transect was in a forested area and the second was in an adjacent open area. Pitfall traps were placed at 10 m intervals (n = 20 per transect). Traps were left open and allowed to collect ants for 48 hours. The samples were taken back to the laboratory where I sorted and identified the specimens using the best available taxonomic key for each genus or subgenus (many of which are now compiled in a recent book “A Field Guide to the Ants of New England” [17]). I counted the number of individuals of each species per trap and converted the abundance matrix to a presence/absence matrix, so that at any given transect, the maximum occurrence of any single species was 20. Incidence data instead of abundance data are commonly used in ant ecology studies to account for the high abundances associated with pitfall traps which happen to be placed close to nests [16]. I estimated richness based on the presence-absence data using the Chao2 and ICE metrics in EstimateS Version 8.2.0 [20]. Voucher specimens will be deposited in the Harvard Museum of Comparative Zoology pending completion of the dissertation research. I present a site by species matrix in File S1.

The paired transects were separated by 500–2000 m. Forested transects were dominated by various overstory species, whereas open transects lacked overstory vegetation and typically had herbaceous and grassy understory vegetation. Samples were collected from May 2010 to August 2010, starting in the southernmost sites and working my way north as the peak growing season advanced. Sites were typically in minimally altered or disturbed state and national forests. Forested transects were selected so that transects started at distances >2 km from major roads and were at least 500 m from the adjacent open transect. Open transects were typically in anthropogenically modified habitat (e.g. power line clearings or pastures).

### Environmental Data and Gradient Models

At each site, I recorded latitude, longitude, and elevation data with a Garmin Oregon 400t (Garmin International Inc. Olathe, Kansas). I extracted data layers for soil, climate, and remotely sensed databases using ArcGIS (version 9.3). I extracted soil information from the United States Department of Agriculture Web Soil Survey [21], climatic variables (mean annual temperature, maximum temperature of the warmest month and minimum temperature of the coldest month and mean annual precipitation) from the WorldClim bioclimatic dataset [22], EVI and NDVI (measures of transect-level productivity) data from the Moderate-Resolution Imaging Spectroradiometer (MODIS) dataset at 250 m resolution for the time period of June-July of 2010 [23] and landcover classification variables from the National Landcover Database (NLCD, [24], File S1). For landcover variables, I calculated the proportion of land classified as forest, developed, agricultural, scrub, wetland and barren in the 1 km^2^ area around the center point of each transect.

At each site I compiled species lists of the main overstory and understory vegetation along the sampling transects. I used principal coordinates analysis (PCoA) to derive site scores based on the vegetation species lists (Figure S1 in File S2). The PCoA scores of the first and second axis for each site were entered as independent variables in stepwise multiple-regression and boosted regression tree (BRT) analyses and treated as measures of vegetation composition at each site.

I checked for colinearity between the temperature and productivity measures and removed variables that were highly correlated (i.e. adjusted *r* ^2^ >0.50); variables removed were: mean annual temperature, minimum temperature of the coldest month and EVI. I used the following variables as independent predictors of observed species richness in the stepwise generalized linear model (GLM) multiple-regression analyses assuming a Poisson link function (which is appropriate for species richness counts) in the MASS package in R (version 2.15) [25], [26]: latitude, longitude, elevation, soil type, maximum temperature of the warmest month, mean annual precipitation, vegetation composition PCoA-1, vegetation composition PCoA-2, NDVI, and the proportions of land around each transect that were classified as forest, developed, agricultural, scrub, wetland and barren. I weighted each observation in the GLM and BRT based on the estimated sampling coverage of each site or transect with using the formula:
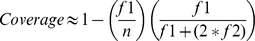
Where *f1* is the number of singletons collected in a transect or site, *f2* is the number of doubletons collected in a transect or site and *n* is the sample size (i.e. number of traps used in the site or transect) [27]. Using this approach places more weight on sites that were more thoroughly sampled and less weight on sites where sampling coverage was lower.

As an alternative to GLMs, I used BRT analysis which is a machine learning approach that can be used to explore the relationships between environmental predictors and response variables using the combination of many simple tree models. BRTs are increasingly used as a species distribution modeling algorithm but can also be applied as a method to explore complex non-linear relationships between environmental attributes and species richness patterns across broad spatial gradients (e.g. predicting patterns of fish species richness in New Zealand [28]). Some of the advantages of using are that BRTs are 1) they allow for the use of non-normally distributed data, 2) categorical data can be used (e.g. soil type), 3) data can be weighted (e.g. using coverage weights), and 4) allow for applying a response variable family distribution (Poisson distribution in this case for count data). Additionally, BRT results are typically more informative than the results of traditional linear modeling approaches [29]. Elith et al. (2008) present a review and explanation of BRT models' usefulness in ecological research [29]. BRTs require that at least two parameters be set, the first is the “tree complexity” which I set at 2 and is appropriate for smaller datasets. Tree complexity reflects the maximum number of allowed nodes in the decision trees of the analysis. The second is the “learning rate” which I set to 0.001 and held constant for all models so that at least 1000 trees would be produced for any given analysis (the smaller the learning rate, the higher the number of trees that can be produced). I used the R package “gbm” to implement this analysis [30].

### Beta-Diversity Analysis

I used the betapart package in R [31], [32] to calculate Sørenson dissimilarity measures and the turnover of species composition between sites. I used the ß_SOR_ metric to evaluate total dissimilarity between sites across the temperature environmental gradient. To account for spatial autocorrelation and correlation between site richness and environmental variables (temperature, precipitation, vegetation composition and productivity) I used a Mantel test on each environmental variable distance matrix against the pair-wise dissimilarity matrix (File S3). I binned the presence/absence data based on the sites' maximum temperature of the warmest month and regressed the corresponding ß_SOR,_ values against temperature to evaluate the differences across the temperature gradient of the northeastern U.S. These regressions are also presented subdivided by ecoregions in File S6. A regression framework for comparing beta dissimilarity values across environmental gradients is appropriate for studies of this geographic scale, magnitude and across environmental gradients [33].

## Results

### Species richness across environmental gradients

**Figure 2:**
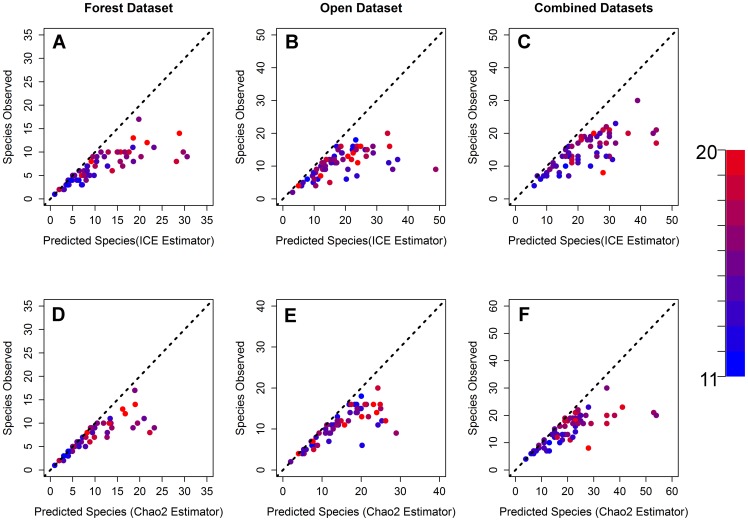
Observed vs. estimated species richness. Scatterplot of observed species richness versus predicted richness using the ICE (A–C) and Chao2 (D–F) estimators for forest and open habitat as well as the combined datasets. Colors of points represent the maximum temperature of the warmest month at each site. The dotted line shows the 1 to 1 relationship between observed and predicted richness.

The transects yielded 16,538 ant specimens representing 92 species. At all sites, richness was higher in the open habitat than in the forest habitat transect (Figure 2). The best sampled transects (i.e. those closest to the 1∶1 ratio line in Figure 2) tended to be in cooler, low richness forested transects (Figure 2A, 2D). Warmer open habitat transects tended to be undersampled (2B, 2E). As many as 30 species were collected at several sites across the extent of the study region (Figure 2C, 2F), but most high diversity sites tended to be along the east coast at warmer sites. The sites along the coastal regions between Massachusetts and Virginia tended to have the highest expected richness (Chao2 estimator ≥50 species of ground foraging ants per site) (Figure 2F). This region of high diversity corresponds to the Southeastern Costal Plains ecoregion. In contrast, the lowest diversity sites (5–10 species) tended to be at high elevations/latitudes and lower temperatures (Figure 2C, 2F), which generally correspond to the Atlantic Highlands ecoregion.

**Figure 3:**
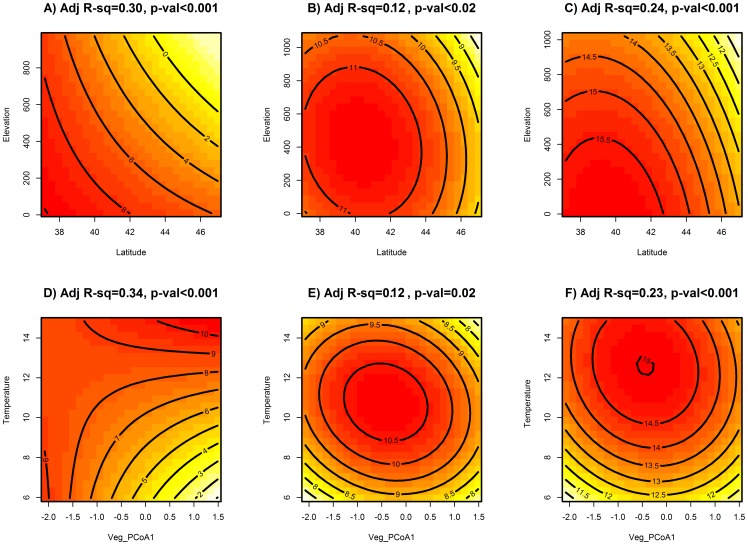
Contour plots of species richness across environmental gradients. Contour plots displaying the predicted species richness based on latitude x elevation interactions (A–C) and maximum temperature of the warmest month X vegetation principal coordinate scores interactions (D–F). A and D are models for the forest dataset only, B and E are models for the open dataset only, and C and F are models for the combined datasets.

In most cases quadratic interactions between latitude and elevation (Figure 3A–C) and temperature and vegetation composition (Figure 3D–F) were the best predictors of species richness. However, the best-fitting models differed for forested and open habitats. Up to 30% of the variation in observed richness in forested habitat was explained by the interaction between latitude and elevation across the spatial gradient (Figure 3A). In open habitats and in the combined dataset (i.e. open habitat + forested habitat), quadratic relationships between latitude and elevation best explained the variation in observed richness, but only accounted for 12% and 24% of the variation in observed species richness respectively (Figure 3B–C).

Results of models using temperature of the warmest month and the best environmental predictor of observed species richness (i.e. the first vegetation principal coordinate score) were similar: a linear model best fit the data in forested habitat (explaining ∼34% of the variation in species richness, Figure 3D) and quadratic interactions best fit observed patterns of richness in open habitat and in the combined datasets (respectively accounting for 12% and 23% of the observed variation in richness across the different sites; Figure 3E–F).

Stepwise variable selection on the GLMs identified the best model for estimating species richness in forest habitats as the one that included latitude, longitude, elevation, the interaction between latitude and elevation, the interaction between longitude and elevation, vegetation principal coordinate score 1and NDVI classification. For open habitats, the best GLM included latitude, longitude, elevation, the interaction between latitude and elevation, the interaction between latitude and longitude, vegetation principal coordinate score 1 and NDVI. When both forest and open habitat data were pooled, the best GLM included latitude, longitude, elevation, the interaction between latitude and elevation, the interaction between longitude and elevation, and the vegetation principal coordinate score 1 (See File S4 for variable contributions on all best GLMs, Table 1).

**Table 1:** Model AIC values for GLMs including all possible predictor variables and the interactions between latitude, longitude and elevations for forests, open and combined datasets.

	Forest Habitat	Open Habitat	Combined Data
*Global Model (All Variables Included)*	304.4	339.28	372.4
*Best Model*	283.4 (V1+V2+V3 +V6+V8+V12+V17+V18)	323.9 (V1+V2+V3+V6+V8+V14 +V16+V17)	361.9 (V1+V2 +V3+V6+V16+V17)
*Effect of Variable Removed From Global Model*			
*V1) Latitude*	307.1*	344.9*	374.1*
*V2) Longitude*	305.4*	340.7*	373.7*
*V3) Elevation*	304.9*	342.3*	374.0*
*V4) Max Temperature of Warmest Month*	302.4	337.3	370.7
*V5) Mean Annual Precipitation*	302.8	337.3	370.5
*V6) Veg. PCoA1*	306.1*	**340.1**	**372.0**
*V7) Veg. PcoA2*	302.4	338.7	370.8
*V8) NDVI*	**303.0**	344.6*	372.4
*V9) Soil Type*	296.1	338.4	367.5
*V10) Proportion of barren land*	304.2	337.5	NA
*V11) Proportion of developed land*	304.3	337.67	NA
*V12) Proportion of forested land*	304.3	337.4	NA
*V13) Proportion of scrub land*	304.7	337.3	NA
*V14) Proportion of agriculture land*	303.9	**337.6**	NA
*V15) Proportion of wetland*	304.0	337.5	NA
*V16) Latitude: Longitude*	305.2	**346.9**	377.0*
*V17) Latitude: Elevation*	301.4*	**342.6**	376.4*
*V18) Longitude: Elevation*	291.6*	339.4	368.9

(*) Indicates significant variable (p≤0.05) contributing to the best model.

I also examined a GLM in which I excluded latitude, longitude, and elevation because these variables often serve as surrogates for abiotic factors such as temperature. In this series of models, the best-fit GLM for forest habitats included the maximum temperature of the warmest month and vegetation principal coordinate score 2 as the best predictors of richness.

For open habitats the best-fit model included the maximum temperature of the warmest month, NDVI and the proportion of agricultural land around the transect. When both forests and open datasets were combined, the best GLM had the maximum temperature of the warmest month, NDVI and Soil Type as the best predictor variables (Table 2).

**Table 2:** Model AIC values for GLMs excluding latitude, longitude and elevation as predictor variables for forests, open and combined datasets.

	Forest Habitat	Open Habitat	Combined Data
*Global Model (All Variables Included)*	306.0	341.5	372.9
*Best Model*	291.9 (V1+V4)	328.9 (V1+V5+V11)	367.7 (V1+V6+V5)
*Effect of Variable Removed From Global Model*			
*V1) Max Temperature of Warmest Month*	324.9*	336.8*	384.5*
*V2) Mean Annual Precipitation*	303.9	339.8	369.6
*V3) Veg. PCoA1*	304.8	339.6	369.5
*V4) Veg. PCoA2*	**293.7**	339.7	369.0
*V5) NDVI*	304.3	**330.12**	**369.9**
*V6) Soil Type*	301.5	340.8	369.7*
*V7) Proportion of barren land*	306.7	339.7	NA
*V8) Proportion of developed land*	306.8	339.7	NA
*V9) Proportion of forested land*	306.7	339.6	NA
*V10) Proportion of scrub land*	307.1	339.7	NA
*V11) Proportion of agriculture land*	306.2	330.9*	NA
*V12) Proportion of wetland*	306.5	339.8	NA

(*) Indicates significant variable (p≤.05) contributing to the best model.

**Figure 4:**
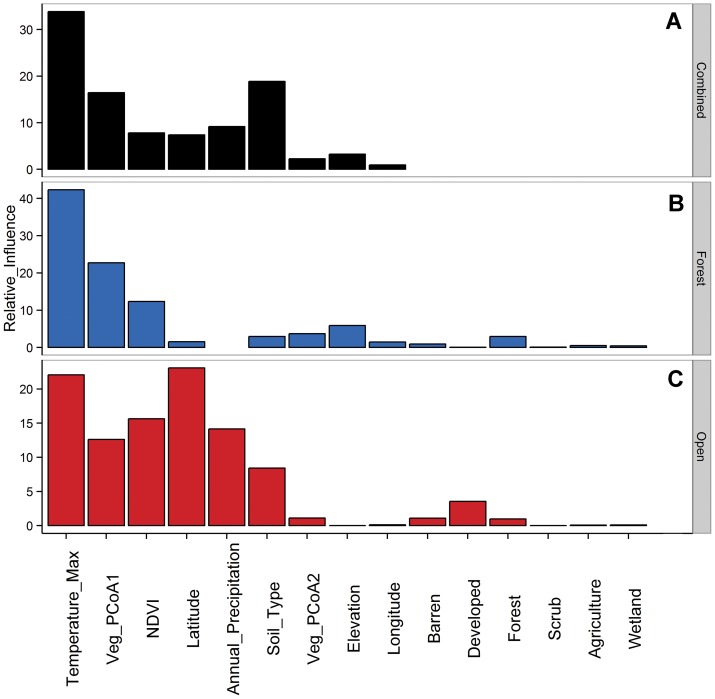
Relative influence of predictor variables in Boosted Regression Tree analyses. A) Black bars indicate variable contributions for the combined data sets, B) Blue for the forest dataset only, and C) red for the open habitat dataset only.

The site level BRT model (i.e. the combined dataset) had a c.v. correlation of 0.43 and explained 35% of the deviance in the data. The best predictors for this model were maximum temperature of the warmest month, the first principal coordinate of vegetation community composition, soil type and mean annual precipitation. The forest habitat model had a c.v. correlation of 0.55 and explained 66% of the deviance in the data and was the best performing of the three BRT models. The model was best predicted by three variables, the maximum temperature of the warmest month, the first principal coordinate of vegetation community composition and NDVI. The open habitat model had a c.v. correlation of 0.15 and explained 25% of the deviance in the data and was the worst performing of the three BRT models. Maximum temperature of the warmest month, latitude, NDVI, the first principal coordinate of vegetation community composition, soil type and annual precipitation were the variables which contributed the most to this model (Table 3, Figure 4). The applied BRT functions and fitted values for each of the models are presented in (File S5).

**Table 3:** Summary of Boosted Regression Tree Models.

Model	Total Deviance	Residual Deviance	Percent of Deviance Explained	Training Data Correlation	C.V. Correlation
Combined Dataset	1.79	1.17	0.35	0.70	0.43
Forest Dataset	1.63	0.56	0.66	0.85	0.55
Open Dataset	1.47	1.10	0.25	0.63	0.15

### Beta-diversity

There were no strong trends of spatial autocorrelation or correlation between the dissimilarities of the environmental variables and the community dissimilarity measures. The most evidence for spatial autocorrelation occurred when the data for open and forest transects were pooled. Sites that were between 29 km and 157 km apart from each other had a Mantel r between 0.05 and 0.07. Sites that were furthest apart from each other (ranging 750 km to 900 km in distance from each other) were negatively correlated Mantel r between −0.17 and −0.11). Slightly more apparent autocorrelations were observed between community dissimilarity and temperature dissimilarity. Once again the strongest evidence for autocorrelation was observed when the forested and open habitat data were pooled. Sites that were similar in mean annual temperatures (±2.2°C) were positively correlated, (Mantel r values ranging from 0.10 to 0.13). Sites that were very different from each other in mean annual temperature measures (±8.6°C) were negatively correlated (Mantel r values ranging from −0.15 and −0.10) (Figure S2 in File S3).

**Figure 5:**
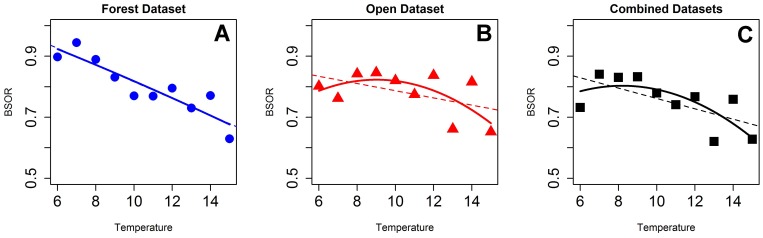
Beta diversity across the temperature gradient. Showing the best-fit linear regression (dashed lines) and quadratic regression (solid lines). Forest habitat (blue circles), open habitat (red triangles) and the combined datasets (black squares). (A–C) Total dissimilarity (ß_SOR_) between sites for Forest (A: R-sq = 0.82, p<0.001) Open (B: R-sq = 0.27, p>0.05) and the combined datasets (C: R-sq = 0.44, p = 0.05).

Beta diversity patterns across temperature gradients differed between forested and open transects. Forested habitat beta dissimilarity values peaked in cooler temperatures and decreased as temperature increased. In contrast, dissimilarity in open habitats and in the combined data set remained fairly constant throughout the temperature gradient and only slightly decreased as temperatures increased to ∼14°C (Figure 5). Linear correlations did not account for any significant percentage of variation explained the relationship between beta dissimilarity and temperature in the combined dataset and the open transects but a strong linear relationship is present in the forest dataset alone (Adjusted R-sq = 0.82, p<0.001, Fig. 5A). The best fit regression for the open transect data was a quadratic relationship between temperature and ß_SOR_ but it was not significant (Adjusted R-sq = 0.28, p>.05, Fig. 5B). A quadratic relationship between temperature and ß_SOR_ was also the best fit for the combined dataset and was statistically significant (Adjusted R-sq = 0.44, p = .05, Fig. 5C). These regressions are subdivided by ecoregions in File S6.

## Discussion

The results presented here demonstrate that alpha and beta diversity of ground-foraging ants in the northeastern United States differ based on habitat type across environmental gradients. Latitudinal gradients of ant species richness previously have been considered for the ants of the northeastern United States [5], [6], [34], where species richness in temperate forests were best predicted by key climatic and local environmental attributes (e.g. mean annual temperature, vegetation community composition, and the presence of the invasive red fire ant [26]). As in these previous studies, the latitudinal patterns of ant species richness in open habitats tended to be weaker than in forested habitats. However, open-habitat species diversity and composition often contribute to general patterns of species richness (Figure 3) and turnover (Figure 4) across regional scales, a pattern that has been observed in other Hymenopteran communities [35] and with ants at finer local scales [36].

One of the variables most useful for predicting species richness in the study region was maximum temperature of the warmest month; it accounted for more of the variation in predicting species richness in forested habitats than in open habitats, perhaps due to the high variability of local temperatures in open habitat. This suggests that mechanisms linked to temperature and climate (e.g. thermal tolerance of species, metabolism) may be regulating species richness and community structure in the temperate forests of the northeastern U.S. In previous studies and at smaller scales [6], assembly rules based on body size also were proposed as being informative in predicting co-occurrences of species in forested plots but were less informative in open habitats. The connection between body size and temperature-related mechanisms was considered for a small subset of species that are common throughout the northeastern U.S. [37], but more work is needed to evaluate how body size and temperature relationships interact across larger species pools and ultimately contribute community assembly patterns. Additional community structuring mechanisms, including interspecific interactions and niche differentiation, were also recently explored at finer local scales, this study also found that ant communities in forests were structured differently from those in open habitats [36].

One of the single variables that best explained the variation in ant species richness across the spatial gradient of the northeastern United States was a measure of vegetation composition. The highest richness values, were observed at sites dominated by deciduous overstory vegetation (e.g. sugar maple, red oak, red maple, or American beech), species-poor sites tended to be dominated by overstory composed of evergreens (hemlock, white pine, spruce or balsam fir). Some exceptions occurred in pine barrens, including Myles Standish State Forest and the various sites in the pine barrens of New Jersey, where species richness was high but the main overstory was composed of pitch pine, long leaf pine and scrub oak. Pine barrens are hotspots of local diversity for other species of plants and arthropods, as well as the ants studied here, but are threatened by management practices and development [38]. Diversity patterns of ants in these hot-spots of richness should be carefully considered in future studies and may reveal patterns that are applicable at larger spatial scales.

Macroecological models have used latitude and elevation as surrogates for describing the climate (mainly temperature) of a given site, but here I have shown that using the maximum temperature of the warmest month as a correlate of richness rather than both latitude and elevation is more informative and yields more biologically relevant information about the correlates and drivers of diversity at the site and transect levels. Using a single measure of temperature (instead of two generally highly correlated variables like latitude and elevation) eliminates the problem of colinearity in analyzing macroecological models in a regression framework and I encourage future studies to consider this in their analyses. Based on the interactions between mean annual temperature and vegetation community composition, it is possible to identify regions of high and low species richness in a simplified framework (Figure 3 D–F), which takes into account biologically meaningful interactions like those between abiotic and biotic correlates of richness (e.g. temperature, and vegetation community composition).

The use of BRT models in this study clearly shows that this machine-learning analytical framework can successfully explain much of the variation in species richness patterns across environmental gradients and in this case was more informative than the traditionally used GLMs. Additionally the results from BRTs are easy to understand and the important variables are clearly identified. I would recommend that future studies continue to use this approach to further explore the complex relationships between abiotic and biotic drivers of species richness patterns.

Ant species richness and turnover changed depending on the ecoregions. The importance of ecoregions on community dissimilarity also needs to be carefully considered when doing studies of regional spatial scales, and has previously been shown to be an important determinant of bird alpha and beta diversity patterns [39] [40] and ant communities of the semi-arid regions of Iran [41]. Not taking into account the impact of ecoregion in the dissimilarity analysis, results in not detecting any significant trend in dissimilarity across the temperature gradient (File S6).

Beta diversity patterns across regional scales also are controlled by geographic and environmental attributes of the landscape [10]. Dissimilarity peaks at cooler temperatures and decreases as temperature increases. This trend was stronger in forested transects, but was also detected when the datasets were pooled. This pattern may be the result of patchy species distributions in cooler regions of the study extent. Decreased dissimilarity in warmer sites may be reflective of more continuous distributions of species across the landscape. At cooler sites, ant species distributions in this region become patchier, likely due to the fact that species are living in thermal extremes. As climate in the region changes, major effects on species distributions and consequently major changes in rates of species turnover are likely to occur (Del Toro, unpublished data). This trend is also reflective of recently described patterns of community dissimilarities of Canadian butterflies where dissimilarity peaks at higher productivity sites and declines as productivity decreases [42]. This pattern contributes to the explanation of why NDVI was normally one of the better predictors of richness in the GLMs and BRTs.

## Conclusions

Communities are changing rapidly as regional climate change impacts the planet and causes changes that have profound ecological and evolutionary consequences [13], [14]. In the ecoregions of the northeastern United States, as temperature increases and as vegetation communities shift from evergreen dominated forests to deciduous forests, ant species richness may increase and dissimilarity between localities of species is likely to decrease. This could potentially result in significant compositional changes (e.g. homogenization of the ant fauna, loss of patchily distributed species, and range expansion of potentially invasive species) in the cooler ecoregions of the forests of the northeastern United States. High elevation sites at lower latitudes may be particularly vulnerable to major changes in community composition of ants, because the unique fauna of these localities is less likely to track changes in temperature due to the sessile nature and limited dispersal potential of ants which may consequently become extirpated. This phenomenon may already be occurring in the high elevation sampling localities of Virginia, Maryland and West Virginia, where community composition and species richness was high and species turnover was low. This observational study contributes as a thorough baseline measure of species richness and community composition of an important and abundant terrestrial arthropod of the forests of the ecoregions of the northeastern United States, which I would encourage to be continuously monitored as climate change impacts the region.

## Supporting Information

File S1 Datasets and Analysis Code.
(ZIP)

File S2 Principal Coordinates Analysis Tables and Figures. Table S1: Site Scores from Principal Coordinates Analysis. Table S2: Vegetation Species Scores from Principal Coordinates Analysis. Figure S1: Site and species biplot of Principal Coordinate Analysis Results. Solid circles indicate site scores presented in table above. Red crosses indicate species scores listed in table above.
(PDF)

File S3 Mantel Test Figure. Figure S2: Mantel Correlogram: Correlations between geographic distance (in km) and mean annual temperature distances (in degrees Celsius). Sorted by forested habitat, open habitat and the combined datasets. Significant correlations are indicated by filled circles (p≤0.05).
(PDF)

File S4 Generalized Linear Model Table Summaries.
(PDF)

File S5 Boosted Regression Tree Fitted Functions and Values. Figure-S3: Combined datasets BRT Fitted Functions for each environmental variable. Figure-S4: Forest dataset BRT Fitted Functions for each environmental variable. Figure-S5: Open dataset BRT Fitted Functions for each environmental variable. Figure-S6: Combined datasets BRT Fitted Values for each environmental variable. Figure-S7: Forest dataset BRT Fitted values for each environmental variable. Figure-S8: Open dataset BRT Fitted values for each environmental variable.
(PDF)

File S6 Ecoregion Beta Diversity Figure. Figure-S9: Beta SIM vs. Temperature regressions subdivided by ecoregion and habitat type.
(PDF)
