# Supplementary material for: Diversity of Eastern North American Ant Communities along Environmental Gradients
Source: PLoS One. 2013 Jul 12;8(7):e67973. doi: 10.1371/journal.pone.0067973 (PMC3709931; doi:10.1371/journal.pone.0067973)
Supplement: File S2 — Principal Coordinates Analysis Tables and Figures. Table S1: Site Scores from Principal Coordinates Analysis. Table S2: Vegetation Species Scores from Principal Coordinates Analysis. Figure S1: Site and species biplot of Principal Coordinate Analysis Results. Solid circles indicate site scores presented in table above. Red crosses indicate species scores listed in table above. (PDF) [file pone.0067973.s002.pdf]

File S2

Site ID	PCoAxis.1 Score	PCoAxis.2 Score
ABSF	-2.077470208	0.22394882
ALLSP	-1.146912936	0.002985527
ANSF	0.932747734	0.302512646
ARSP	0.469375482	-0.35915954
BAXP	0.597013691	0.749807201
BBSP	-0.055318592	-0.887767641
BESF	-0.829018399	-0.42130299
BTBSP	-0.837559346	-0.177772515
CFSP	-0.122757279	-1.213041239
CHESP	-0.1635147	-0.512999686
CHSP	0.743801748	-0.299039236
COKSP	0.151564943	-0.617231691
CONROB	-1.178976488	1.274315532
CRKSP	0.217819693	1.007587043
CSF	-2.077470208	0.22394882
CWSF	-0.473169107	0.59943944
DRRD	-0.262474592	1.407808043
ENSF	-0.473169107	0.59943944
FBSF	0.211980884	0.623349573
FBSP	1.196393066	-0.339462659
GASF	0.755339367	0.063931206
GBSF	0.186820933	1.148916087
GFSP	-1.418853446	0.190166399
GMNF1	1.316981529	0.349241479
GMNF2	1.018830792	0.297886164
GMNF3	1.251453314	0.463047017
GPRD	-0.77989005	0.84187424
GRRSF	-0.39491938	0.961383621
GWSF	1.269641443	0.2587362
HFLTER	-0.140301531	-0.799481902
HFLTER2	-0.375050376	-1.535425771
HPSF	-0.444474563	-0.610526241
LACSF	-0.209737644	-0.898550232
LRD	-1.169415652	-0.440313574

Site ID	PCoAxis.1 Score	PCoAxis.2 Score
LRSP	-0.39330574	0.34302629
LSF	-0.864116928	0.768479416
MMSP	-0.383496254	-1.248736456
MOHO	0.246455324	0.194380038
MPSP	0.955901006	-0.42860405
MSSP	-0.405651646	-0.443195224
MTCAR	1.148823951	0.035905751
MTGSR1	1.485655153	0.516540154
MTGSR2	0.150793409	-0.082394643
NATSF	1.001955646	-0.339383181
PACSF	0.341914612	-0.809854542
PESF	-1.348785824	0.674220869
PGSP	0.524039362	0.042236304
PITSF	0.604905703	0.674694619
PRSF	-0.875514563	0.11752935
QUESP	1.066702526	0.090017955
RAMT	-0.136641731	-0.599354719
RBSP	-0.246757804	-0.723647661
RSF	-0.964246375	-0.362157507
SPRSF1	0.530274444	0.311621198
SPRSF2	0.632035519	0.421970656
SRISP	0.41410239	0.559214418
STKSF	-1.046140742	-0.514915054
WEISF1	-0.021644149	-0.392047674
WEISF2	0.11808008	0.262647043
WFSP	0.245647854	0.580219206
WILSF	0.293658434	-1.173720405
WMNF1	0.29434573	-0.885654789
WMNF2	1.347004565	0.42909912
WMNF3	0.149383773	-1.179419909
WOMSP	1.155382145	-0.073176695
WSF	-0.41527783	0.880883461
WSRD	-0.366122075	0.485232016

File S2- Table 1: Site Scores from Principal Coordinates Analysis

Species	Score1	Score2
Abibal	0.155917	0.002305
Acepen	0.027631	0.027612
Acerrub	-0.36515	-0.27663
Acesac	0.579522	0.384929
Acespi	0.040773	0.010512
Ambra	-0.03786	0.051772
Aritri	0.023886	-0.01215
Astspp	-0.01707	0.014708
Aurnud	0.073278	-0.01238
Betall	0.36615	0.107278
Betcor	0.004843	-0.00335
Betlen	-0.22574	0.013785
Betpap	0.061086	-0.09981
Bryspp	0.025499	0.041118
Carcar	-0.05082	-0.01312
Cargla	-0.00525	-0.02084
Carova	-0.23871	0.023529
Carspp	-0.07342	0.312176
Cartom	-0.22194	0.042732
Chathy	-0.03689	-0.05386
Chimac	-0.09958	0.000284
Cirspp	0.029954	0.01229
Clealn	-0.03683	0.000121
Clibor	0.004843	-0.00335
Comspp	0.072109	-0.02908
Copspp	0.036268	-0.02197
Corflo	-0.04556	0.007726
Corspp	-0.02891	0.10481
Cryspp	0.040773	0.010512
Daucar	0.043434	0.062178

Species	Score1	Score2
Faggra	0.435666	0.423908
Frapen	-0.13343	0.018197
Fraves	0.038598	0.057874
Gaupro	0.013566	0.001696
Gerrob	0.040773	0.010512
Hamvir	-0.02248	0.023658
Ilespp	-0.04556	0.007726
Junvir	-0.17675	0.045589
Kallat	-0.15493	-0.1328
Liqspp	-0.04331	0.029129
Lirtul	-0.16938	0.034038
Lycspp	-0.00522	-0.01279
Miacan	0.207264	-0.14679
Mitrep	-0.01263	0.013936
Narspp	0.006808	0.025325
Nysspp	-0.05759	-0.03183
Parqui	-0.2598	0.094505
Phespp	-0.02812	0.004775
Picgla	0.092081	-0.02222
Picrub	0.081431	0.009067
Pinres	0.003021	-0.08398
Pinrig	-0.03783	-0.06877
Pinstro	0.008416	-0.62559
Pintae	-0.04331	0.029129
Pinvir	-0.02505	0.034203
Poaspp	0.114352	-0.07272
Polcom	0.025162	0.030528
Potspp	-0.03097	-0.01471
Prupen	0.006808	0.025325
Pruser	0.010538	0.034209

Species	Score1	Score2
Ptespp	0.155513	-0.09405
Quealb	-0.35082	0.206518
Queber	-0.23736	-0.10335
Quecoc	0.008047	0.001878
Quepal	-0.00525	-0.02084
Quepri	-0.18664	0.235225
Querub	-0.49804	0.209185
Ranspp	-0.04556	0.007726
Rosmul	-0.04556	0.007726
Rubale	-0.04598	0.039307
Ruball	-0.14767	0.15393
Rubhis	-0.31339	0.202092
Rubide	0.006808	0.025325
Rubleu	-0.03097	-0.01471
Rubspp	0.439035	-0.14288
Sasalb	-0.07521	0.08602
Smispp	-0.0137	0.026845
Solspp	0.107291	0.013583
Sumspp	-0.00439	-0.02435
Tarspp	-0.0336	-0.02092
Toxrad	-0.13796	-0.08063
Tribor	0.213228	-0.01104
Trilspp	0.014589	-0.02639
Trispp	0.061581	-0.08252
Tsucan	0.31166	-0.35099
Vacang	-0.03683	0.000121
Vaccor	-0.03683	0.000121
Vacspp	-0.2006	-0.15461
Viblan	0.048662	0.034085
Viospp	-0.00898	-0.05556

File S2- Table 2: Vegetation Species Scores from Principal Coordinates Analysis

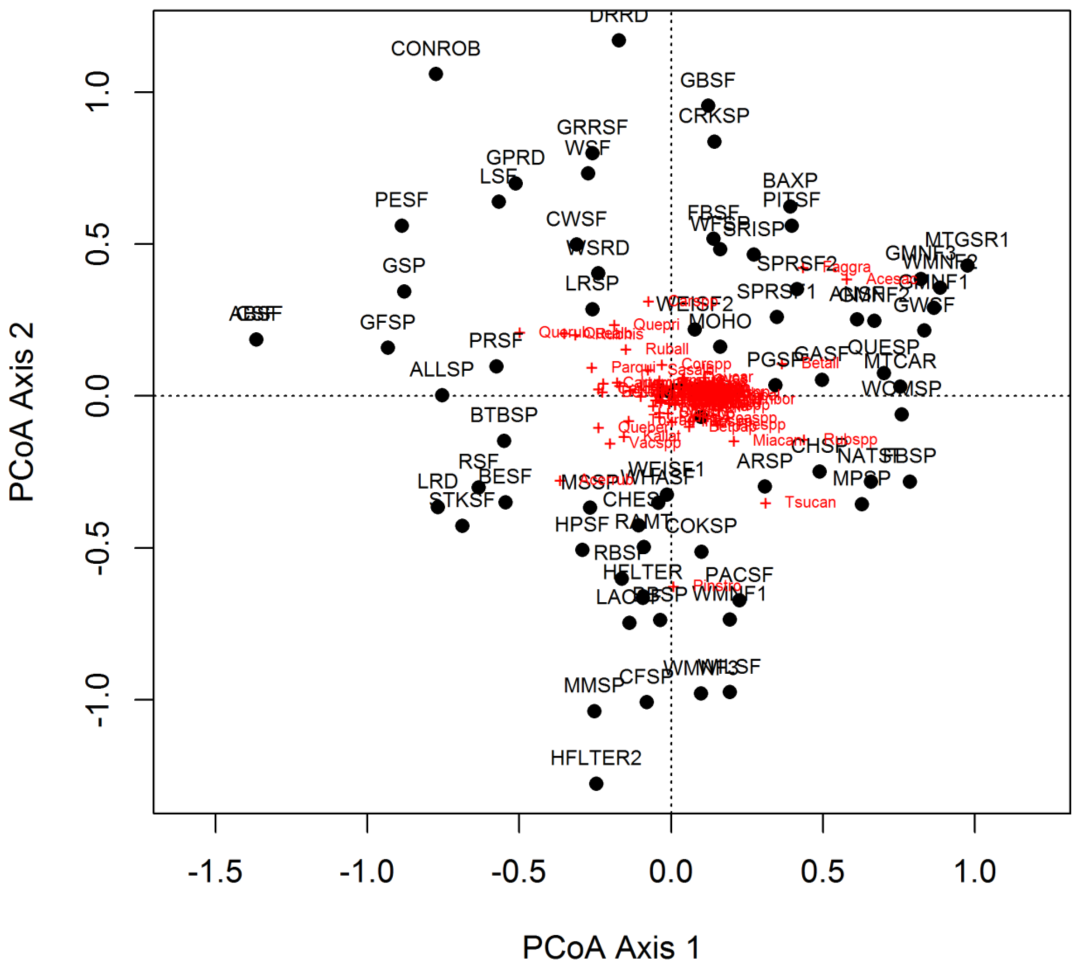

S2- Figure 1: Site and species biplot of Principal Coordinate Analysis Results. Solid circles indicate site scores presented in table above. Red crosses indicate species scores listed in table above.
