# Supplementary material for: Diversity of Eastern North American Ant Communities along Environmental Gradients
Source: PLoS One. 2013 Jul 12;8(7):e67973. doi: 10.1371/journal.pone.0067973 (PMC3709931; doi:10.1371/journal.pone.0067973)
Supplement: File S3 — Mantel Test Figure. Figure S2: Mantel Correlogram: Correlations between geographic distance (in km) and mean annual temperature distances (in degrees Celsius). Sorted by forested habitat, open habitat and the combined datasets. Significant correlations are indicated by filled circles (p≤0.05). (PDF) [file pone.0067973.s003.pdf]

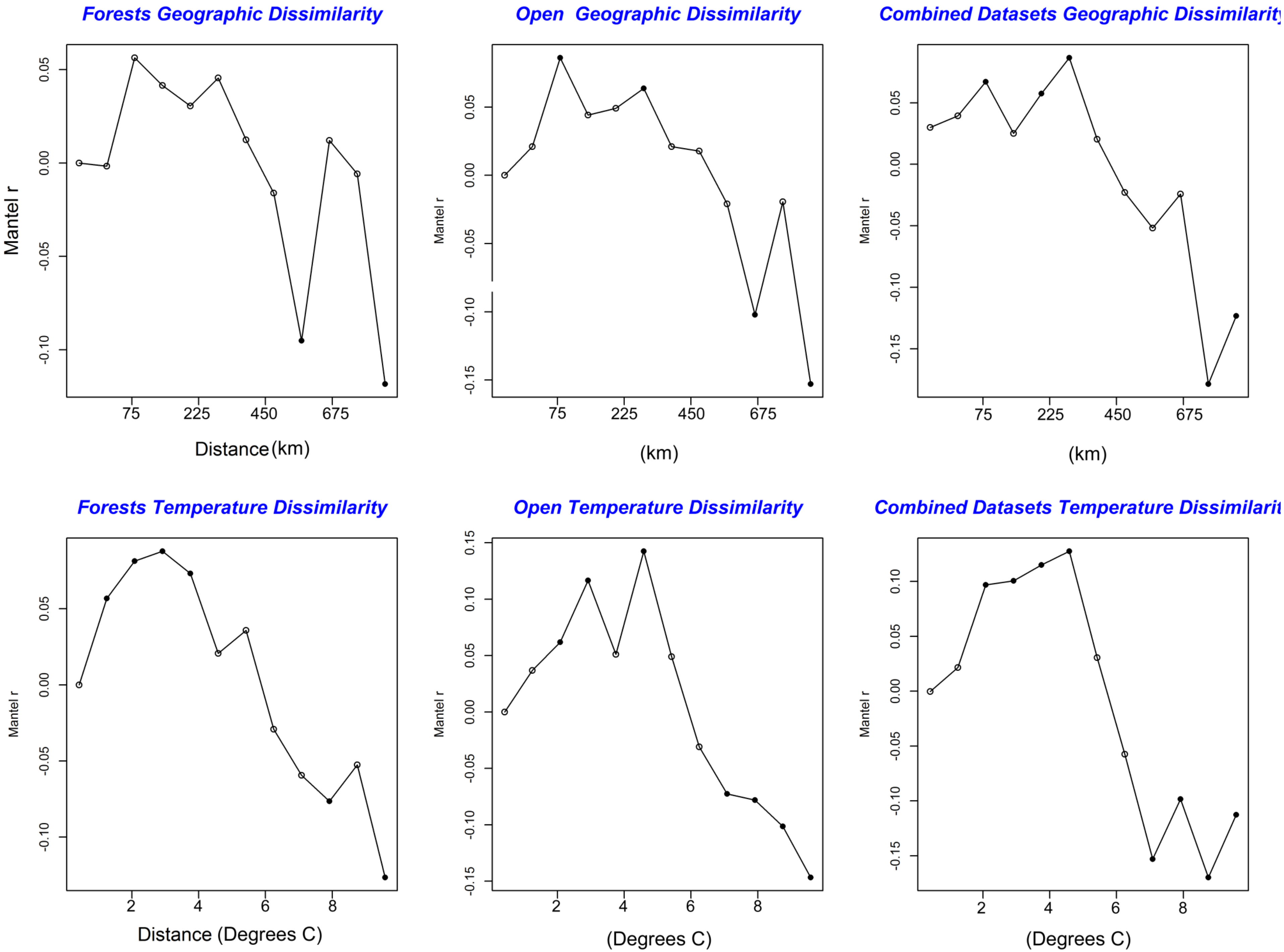

File S3: Figure 1: Mantel Correlogram: Correlations between geographic distance (in km) and mean annual temperature distances (in degrees Celsius). Sorted by forested habitat, open habitat and the combined datasets. Significant correlations are indicated by filled circles (p≤0.05).
