# Supplementary material for: Diversity of Eastern North American Ant Communities along Environmental Gradients
Source: PLoS One. 2013 Jul 12;8(7):e67973. doi: 10.1371/journal.pone.0067973 (PMC3709931; doi:10.1371/journal.pone.0067973)
Supplement: File S4 — Generalized Linear Model Table Summaries. (PDF) [file pone.0067973.s004.pdf]

File S4: Generalized Linear Model Table Summaries

Model details associated with Table 1

Model:
glm(formula = Species_Observed ~ Latitude + Longitude + Elevation + Veg_PCoA1 + NLCD_Name + Latitude:Elevation, family = "poisson")

Variable	Estimate	Standard Error	p-value
Intercept	18.2055674	7.0987605	0.01033
Latitude	-0.1344350	0.0787792	0.08792
Longitude	0.1467120	0.0581913	0.01170
Elevation	0.0095586	0.0043442	0.02778
Veg PCoA1	-0.2896775	0.1023921	0.00467
NLCD Name	0.2501576	0.1503892	0.09623
Latitude:Elevation	-0.0002254	0.0001068	0.03493

Model:
glm(formula = Species_Observed ~ Latitude + Longitude + Elevation + Veg_PCoA1 + NDVI + Latitude:Elevation + Latitude:Longitude, family = "poisson")

Variable	Estimate	Standard Error	p-value
Intercept	7.610e+01	2.134e+01	0.000363
Latitude	-1.789e+00	5.011e-01	0.000358
Longitude	1.032e+00	2.889e-01	0.000356
Elevation	1.100e-02	3.943e-03	0.005264
Vegetation PCoA1	-1.499e-01	7.851e-02	0.056212
NDVI	2.009e+00	7.717e-01	0.009249
Latitude*Longitude	-2.454e-02	6.871e-03	0.000356
Latitude*Elevation	-2.709e-04	9.675e-05	0.005104

Model:
glm(formula = Species_Observed ~ Latitude + Longitude + Elevation + Veg_PCoA1 + Latitude:Elevation + Latitude:Longitude)

Variable	Estimate	Standard Error	p-value
Intercept	6.521e+01	1.768e+01	0.000226
Latitude	-1.404e+00	4.141e-01	0.000696
Longitude	8.089e-01	2.391e-01	0.000717
Elevation	8.700e-03	3.235e-03	0.007163
Veg PCoA1	-1.014e-01	6.535e-02	0.120840
Latitude*Longitude	-2.139e-04	7.913e-05	0.006882
Latitude*Elevation	-1.816e-02	5.690e-03	0.001416

Model details associated with Table 2

Model:
glm(formula = Species_Observed ~ Soil_Type + Veg_PCoA1 + NLCD_Name)

Variable	Estimate	Standard Error	p-value
Intercept	0.56512	0.73048	0.439147
Soil Type	1.56023	0.75369	0.038442
Veg PCoA1	-0.30571	0.07414	3.74e-05
NLCD Class	0.51616	0.14503	0.000372

Model:
glm(formula = Species_Observed ~ Soil_Type + Veg_PCoA1 + NDVI)

Variable	Estimate	Standard Error	p-value
Intercept	0.09462	0.69179	0.89121
Soil Type	0.57896	0.21508	0.00711
Veg PCoA1	-0.13158	0.05228	0.01184
NDVI	2.58105	0.81742	0.00159

Model:
glm(formula = Species_Observed ~ Soil_Type + Temperature_Max + NDVI)

Variable	Estimate	Standard Error	p-value
Intercept	0.665471	0.610866	0.2760
Soil Type	0.336299	0.178069	0.0589
Temp Max	0.064303	0.014670	1.17e-05
NDVI	1.142171	0.633106	0.0712
