# Supplementary material for: Diversity of Eastern North American Ant Communities along Environmental Gradients
Source: PLoS One. 2013 Jul 12;8(7):e67973. doi: 10.1371/journal.pone.0067973 (PMC3709931; doi:10.1371/journal.pone.0067973)
Supplement: File S6 — Ecoregion Beta Diversity Figure. Figure-S9: Beta SIM vs. Temperature regressions subdivided by ecoregion and habitat type. (PDF) [file pone.0067973.s006.pdf]

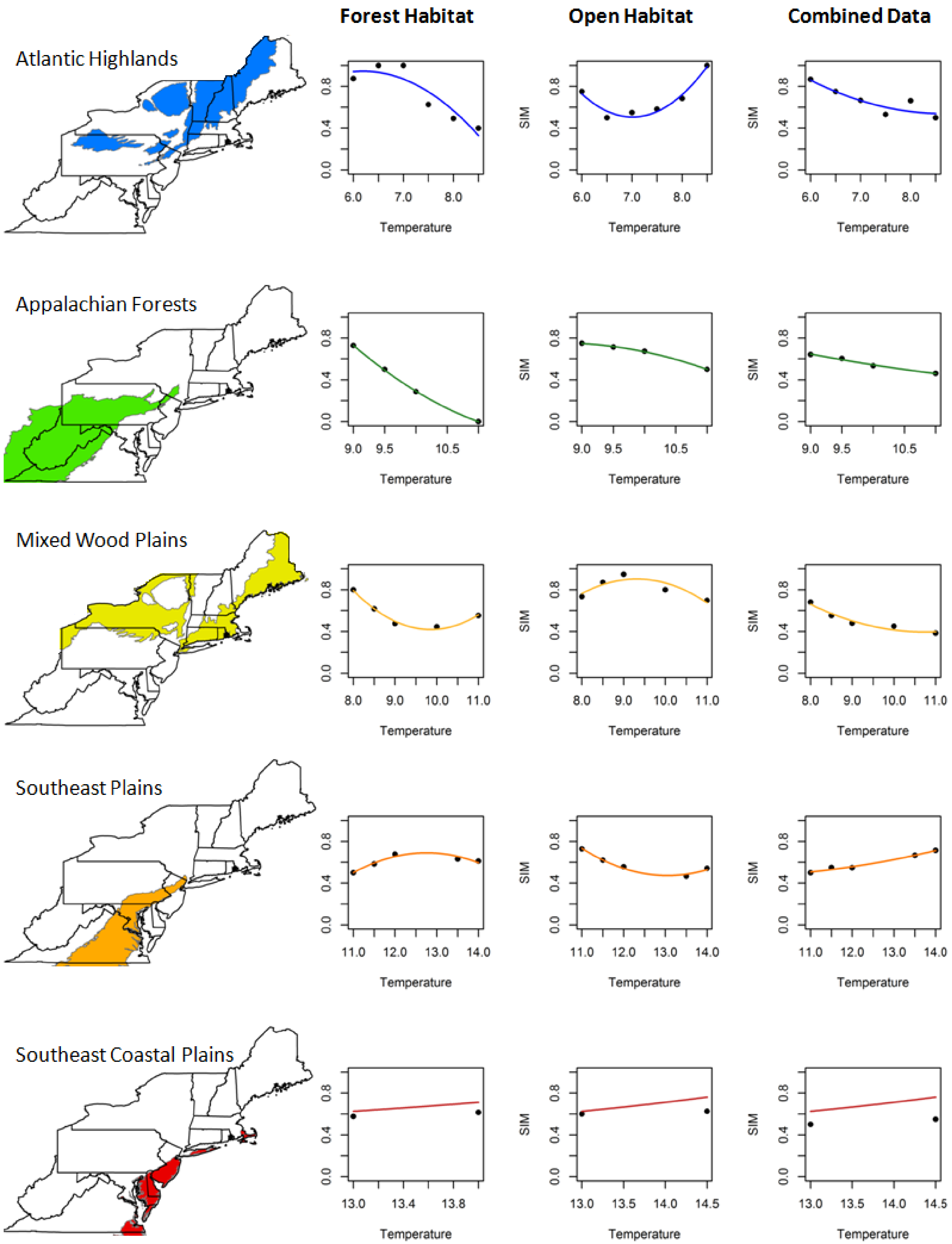

File S6 Figure S9: Beta SIM vs. Temperature regressions subdivided by ecoregion and habitat type
