# Supplementary material for: Diversity of Eastern North American Ant Communities along Environmental Gradients
Source: PLoS One. 2013 Jul 12;8(7):e67973. doi: 10.1371/journal.pone.0067973 (PMC3709931; doi:10.1371/journal.pone.0067973)
Supplement: File S5 — Boosted Regression Tree Fitted Functions and Values. Figure-S3: Combined datasets BRT Fitted Functions for each environmental variable. Figure-S4: Forest dataset BRT Fitted Functions for each environmental variable. Figure-S5: Open dataset BRT Fitted Functions for each environmental variable. Figure-S6: Combined datasets BRT Fitted Values for each environmental variable. Figure-S7: Forest dataset BRT Fitted values for each environmental variable. Figure-S8: Open dataset BRT Fitted values for each environmental variable. (PDF) [file pone.0067973.s005.pdf]

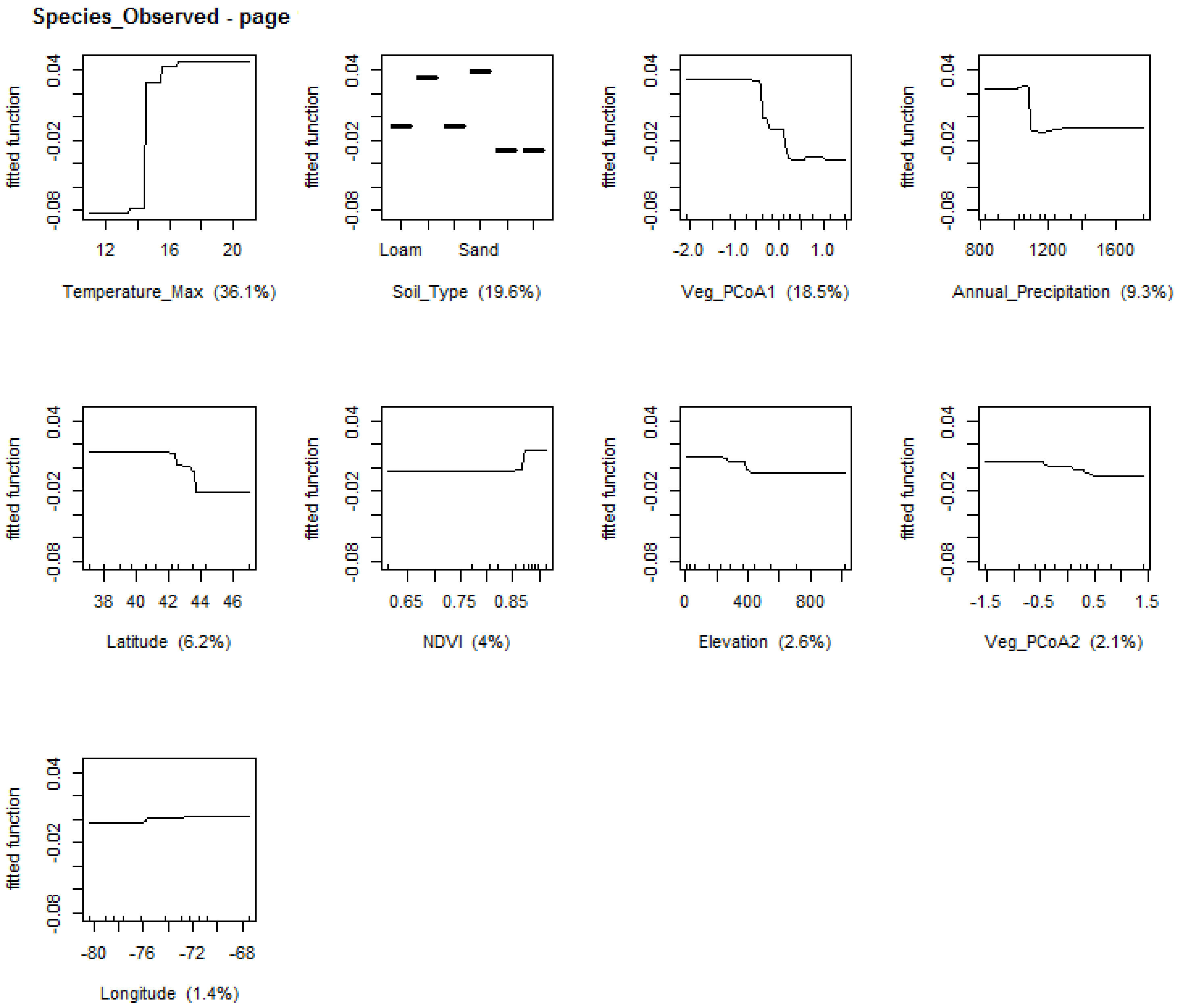

File S5 Figure S3: Combined datasets BRT Fitted Functions for each environmental variable

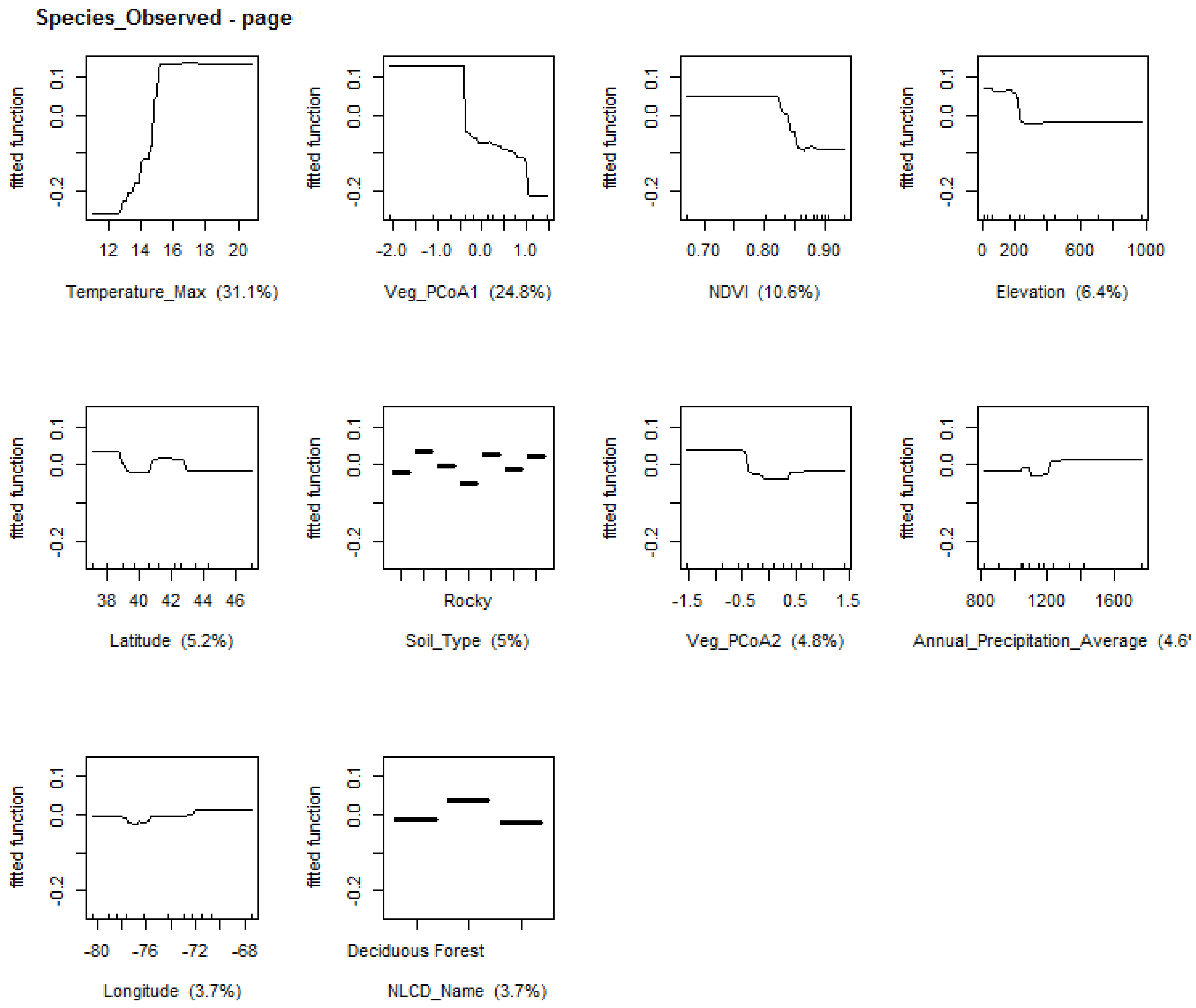

File S5 Figure-S4: Forest dataset BRT Fitted Functions for each environmental variable

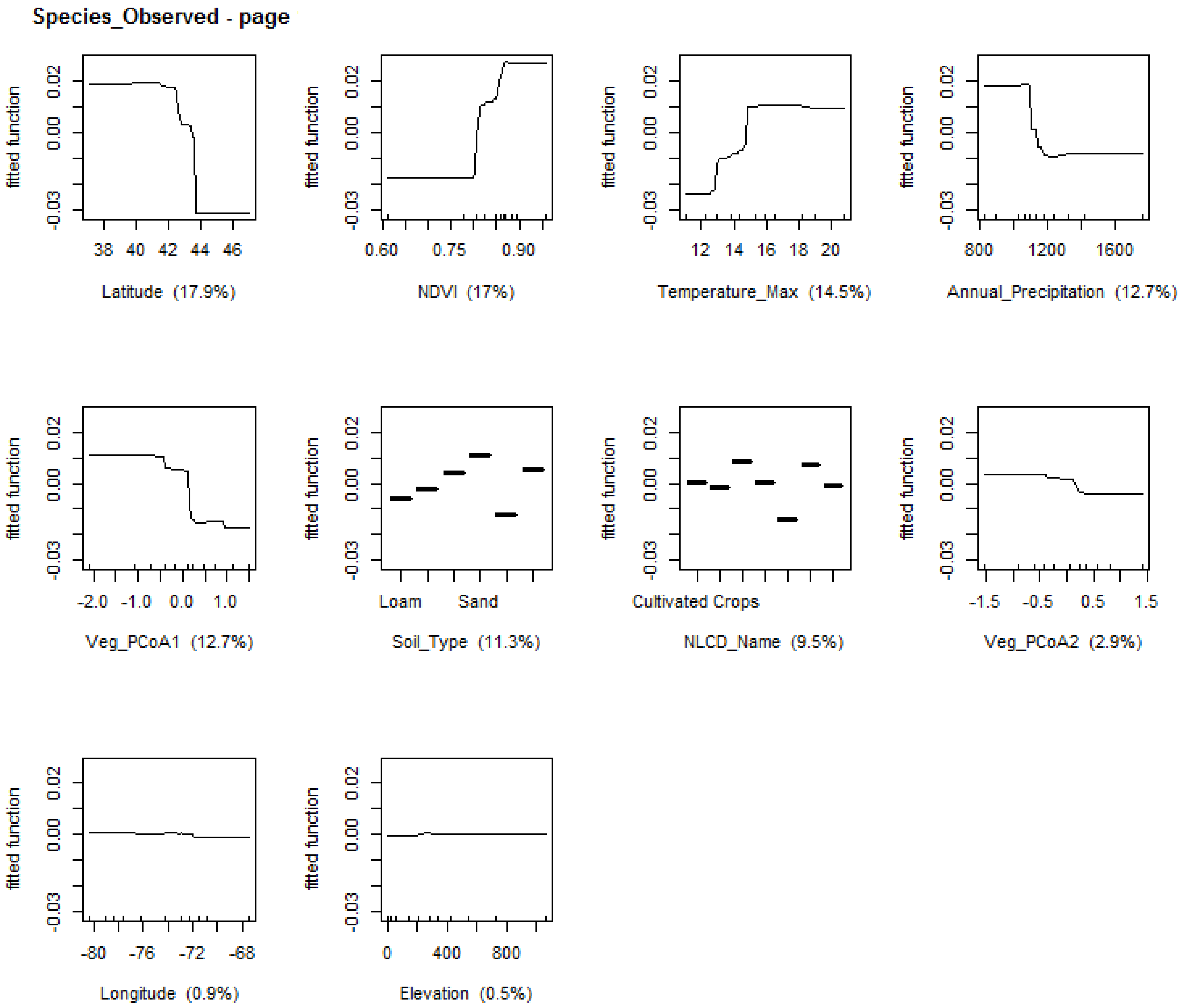

File S5 Figure S5: Open dataset BRT Fitted Functions for each environmental variable

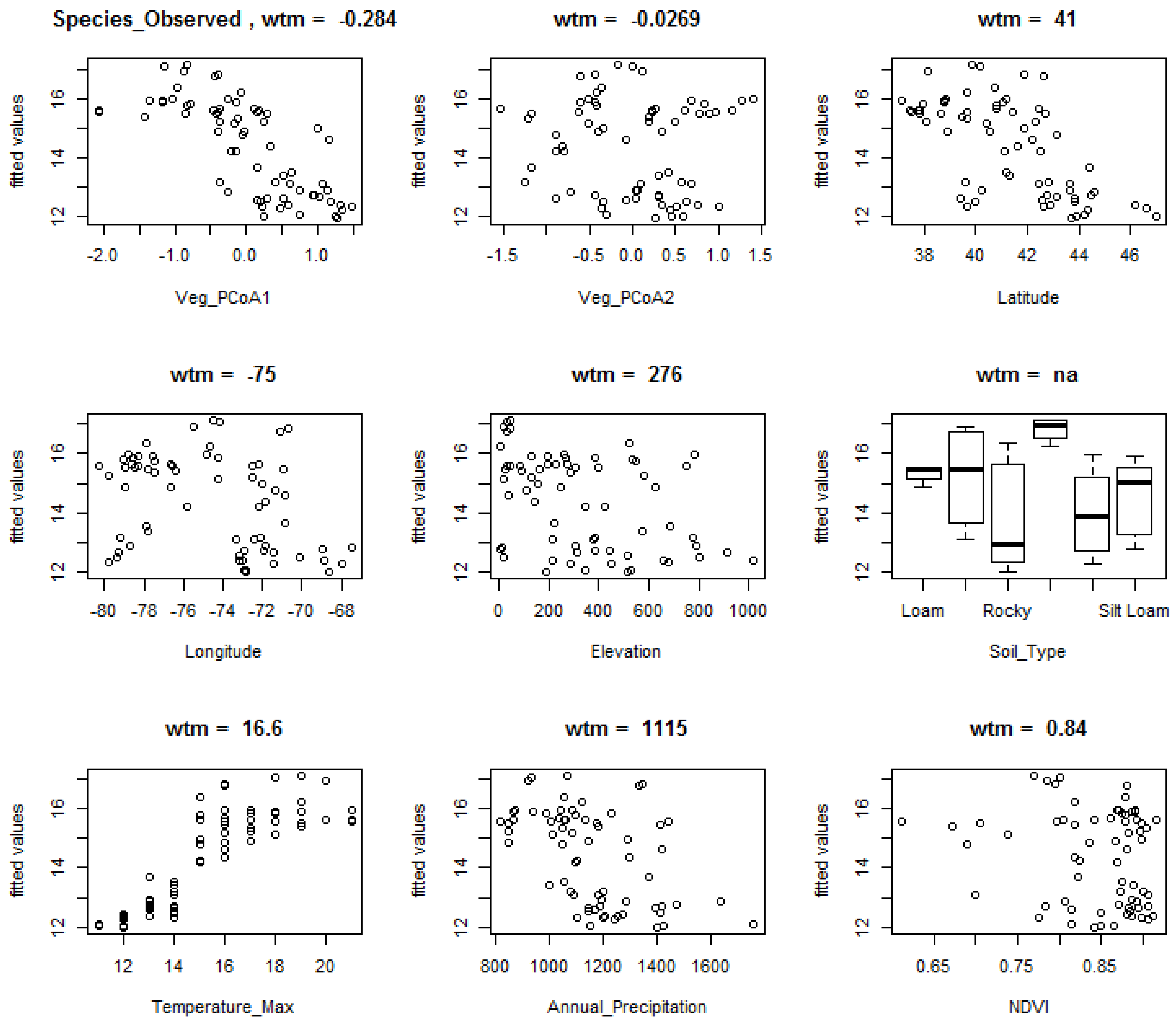

File S5 Figure S6: Combined datasets BRT Fitted Values for each environmental variable

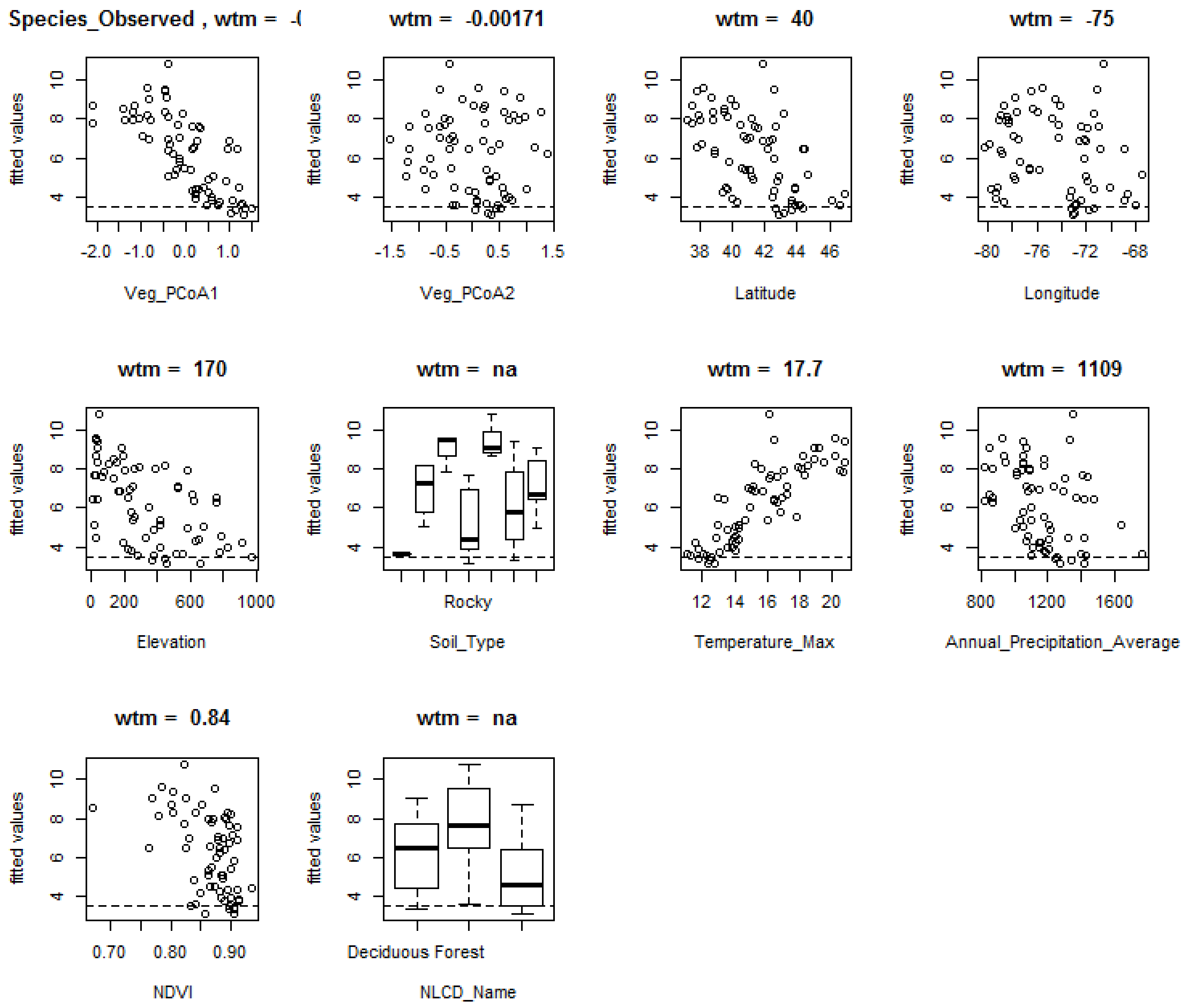

File S5 Figure S7: Forest dataset BRT Fitted values for each environmental variable

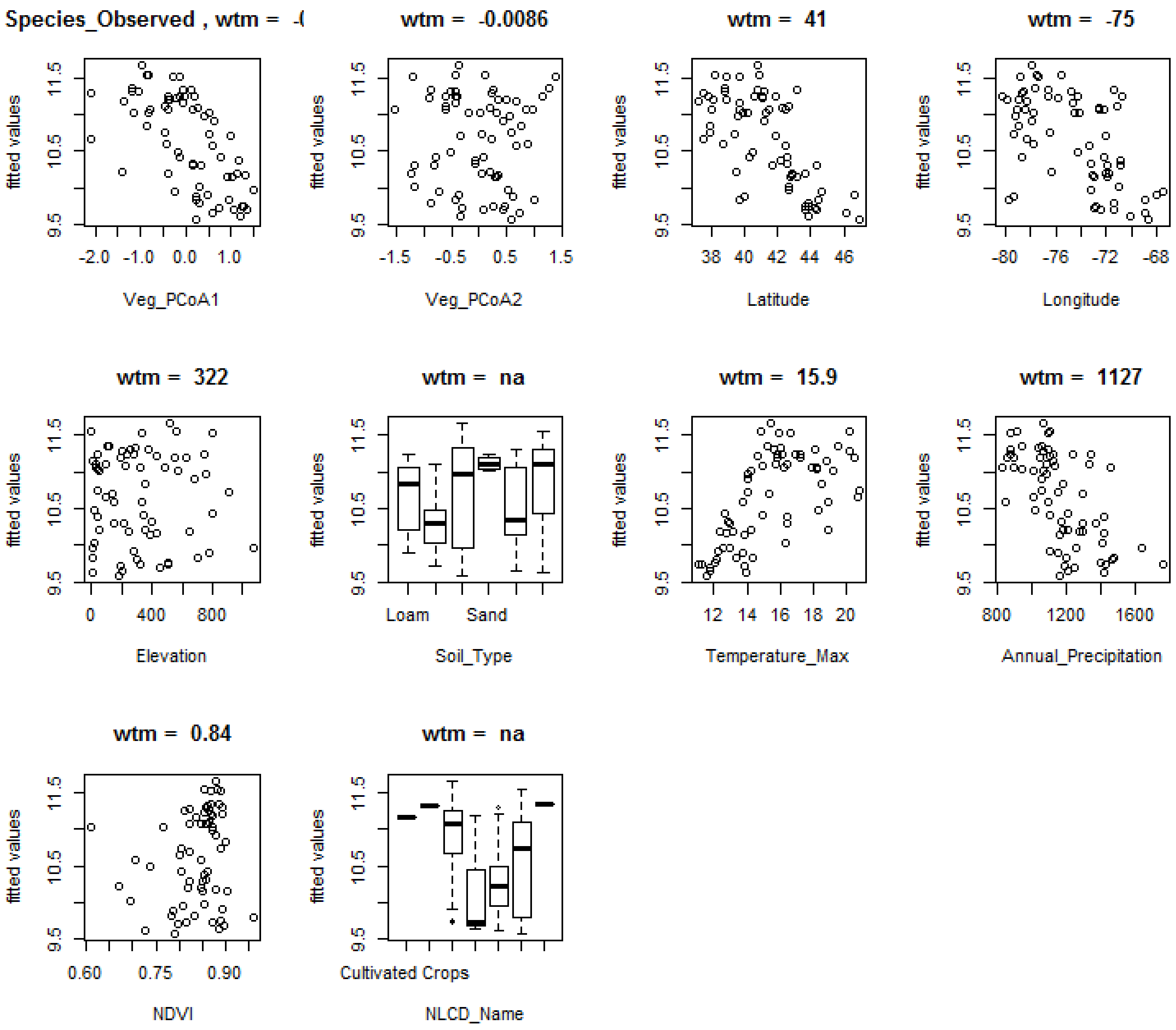

File S5 Figure S8: Open dataset BRT Fitted values for each environmental variable
